# Associations between COVID-19 infection and sex steroid hormones

**DOI:** 10.3389/fendo.2022.940675

**Published:** 2022-10-11

**Authors:** Zixin Cai, Jiaxin Zhong, Yingling Jiang, Jingjing Zhang

**Affiliations:** ^1^ National Clinical Research Center for Metabolic Diseases, Metabolic Syndrome Research Center, Key Laboratory of Diabetes Immunology, Ministry of Education, and Department of Metabolism and Endocrinology, The Second Xiangya Hospital of Central South University, Changsha, China; ^2^ Department of Metabolism and Endocrinology, The Affiliated Zhuzhou Hospital Xiangya Medical College CSU, Zhuzhou, China

**Keywords:** COVID-19, sex hormones, meta-analysis, predict, WMD

## Abstract

**Aims:**

Coronavirus disease 2019 (COVID-19) is caused by infection with severe acute respiratory syndrome coronavirus 2 (SARS-CoV-2), and within a few months of the first outbreak, it was declared a global pandemic by the WHO. The lethal virus SARS-CoV-2 is transmitted through respiratory droplets and enters host cells through angiotensin-converting enzyme 2 (ACE-2) receptors. ACE-2 receptors are highly expressed in many tissues, including testes. Therefore, the objective of this study was to summarize the available literature regarding the correlation between sex hormone levels and COVID-19.

**Methods:**

The PubMed, Web of Science, Embase, and Cochrane Library databases were reviewed systematically through August 2022 for studies comparing sex hormone levels between different patient groups: COVID-19 versus no COVID-19, more severe versus less severe COVID-19, and non-survivors versus survivors. Various types of clinical research reporting sex hormone levels, including free testosterone (FT), luteinizing hormone (LH), follicle-stimulating hormone (FSH), 17β-oestradiol (E_2_), the oestradiol-to-testosterone ratio (E_2_/T), prolactin (PRL), and sex hormone-binding globulin (SHBG), were included. Random- or fixed-effects models were used to calculate weighted mean differences (WMDs) and 95% confidence intervals (CIs). Heterogeneity among the studies was assessed by the *I^2^* index, and data analyses were performed using meta-analysis with Stata version 12.0.

**Results:**

Twenty-two articles that included 3369 patients were ultimately included in the meta-analysis. According to analysis of the included studies, patients with COVID-19 had significantly low T/LH, FSH/LH, and SHBG levels and high levels of LH, and E_2_/T, but their levels of FT, FSH, PRL, E_2_, and progesterone were not affected. Publication bias was not found according to funnel plots and Egger’s regression and Begg’s rank correlation tests.

**Conclusion:**

Low T/LH, FSH/LH, and SHBG serum levels and high LH, and E_2_/T levels may increase the risk of COVID-19. Additionally, the greater is the clinical severity of COVID-19, the higher is the probability of increases in LH, and E_2_/T serum levels and decreases in T/LH, FSH/LH, and SHBG levels. COVID-19 may have unfavourable effects on gonadal functions, which should be taken seriously by clinicians. Routine monitoring of sex hormone levels might help clinicians to evaluate disease severity in patients with COVID-19.

## Introduction

In early December 2019, the outbreak of coronavirus disease 2019 (COVID-19) quickly progressed to a pandemic, bringing severe challenges to global health; this disease can be transmitted through respiratory droplets or by direct contact (1). As of September 8th, 2021, there were more than 4,582,338 deaths (2). In addition, vulnerable individuals may experience a variety of serious and life-threatening complications, including acute respiratory distress syndrome (ARDS), sepsis, coagulopathy, disseminated intravascular coagulation (DIC), acute kidney injury (AKI), and multiorgan dysfunction (3–5). Thus, a better understanding of clinical risk factors that can distinguish between severe and non-severe cases or between a high and low risk of death is vital for improving therapeutic interventions.

Currently, studies have indicated that sex differences are present in the clinical outcomes of COVID-19 patients (6). Sex disaggregated data indeed showed that men had higher rates of mortality than women (7). In particular, the mortality rates of men are 2.4 times higher than those of women, thus indicating sex-specific differences in severe acute respiratory syndrome coronavirus 2 (SARS-CoV-2)-associated sequelae (8). Angiotensin-converting enzyme 2 (ACE2) and TMPRSS2 are critical factors for virus transmission. SARS-CoV-2 is mainly transmitted *via* respiratory droplets and enters human cells through ACE-2 receptors (9). In addition, expression pattern analysis of ACE2 in adult human testes indicated that ACE2 is mainly distributed in spermatogonia and Sertoli and Leydig cells, suggesting that the human testis is susceptible to SARS-CoV-2 (10). TMPRSS2 is predominantly expressed in spermatogonia and spermatids. In a retrospective study, a large proportion of the total patients with COVID-19 were male (11). Consequently, concern has been raised about whether SARS-CoV-2 may affect the male reproductive system. It is assumed that SARS-CoV-2 may have a negative impact on the male reproductive tract; however, results are inconsistent.

## Method

### Search strategy

To find all studies that evaluated the association between sex hormone levels and the risk of COVID-19, two of the authors (ZC and JZ) independently searched the PubMed, Embase, Web of Science, and Cochrane Library databases through August 2022. The search terms included the following key words: (a) COVID-19; (b) testosterone (FT), luteinizing hormone (LH), follicle-stimulating hormone (FSH), 17β-oestradiol (E_2_), the oestradiol-to-testosterone ratio (E_2_/T), prolactin (PRL), progesterone and sex hormone-binding globulin (SHBG). The reference lists of relevant reviews or included studies were also manually searched to identify relevant articles.

### Study selection

Using the PECO/PICO (population, exposure/intervention, comparison/control, and outcome) strategy, we included the studies that met the following criteria in the study.

- Populations: subjects participating in studies that assessed the impact of sex hormone levels on COVID-19.
- Exposure/Intervention: presence or absence of COVID-19
- Comparison: sex hormone levels
- The outcome of the study: sex hormone levels
- Exclusion criteria
- Studies without full text
- *In vitro* and animal studies
- Data of interest were not presented
- Abstracts, commentary articles, reviews, meta-analyses, editorials and conference presentations

### Data extraction and quality assessment

Two of us (ZC, JZ) independently extracted the data using a standardized data extraction form. Extracted information included the following: (1) the characteristics of the study, including the first author, year of publication, study design, and country; (2) basic characteristics of the population, including the sample size, mean age, and sex ratio; and (3) sex hormone indicators, including FT, LH, FSH, E_2_, E_2_/T, PRL, progesterone and SHBG. The weighted mean difference (WMD) and corresponding 95% confidence intervals (CIs) as well as *I^2^* were extracted. For studies reporting only the median ± interquartile range (IQR), we converted these values into the mean and standard deviation (SD) (12). Any dissenting opinions were resolved through discussion and consensus. Quality assessment of the nonrandomized comparative studies was performed with the Newcastle–Ottawa scale (NOS) (13).

### Statistical analysis

Statistical analyses were carried out by STATA (Version 12.0; STATA Corporation, College Station, TX, USA) software. Fixed-effects or random-effects models were adopted according to the heterogeneity of the studies (*I^2^* < 50%, fixed-effects models; *I^2^* > 50%, random-effects models). The WMD with the random-effects model (DerSimonian–Laird method) and 95% CI were applied for continuous data. Sensitivity analysis was conducted by excluding one study each time through influence analysis to assess the stability of the results. Heterogeneity among the included studies was assessed with the *I* ^2^ statistic. *I* ^2^ values above 70% were considered to indicate the presence of extreme heterogeneity. The potential evidence of publication bias was assessed using a funnel plot, Egger’s regression and Begg’s rank correlation tests. If publication bias was confirmed, it was corrected using Duval’s trim-and-fill method used the properties of the funnel plot. Subgroup analysis was conducted based on the severity of the disease or age and country. Statistical significance was determined with a two-tailed *p* < 0.05.

## Results

### Study selection

**Figure 1:**
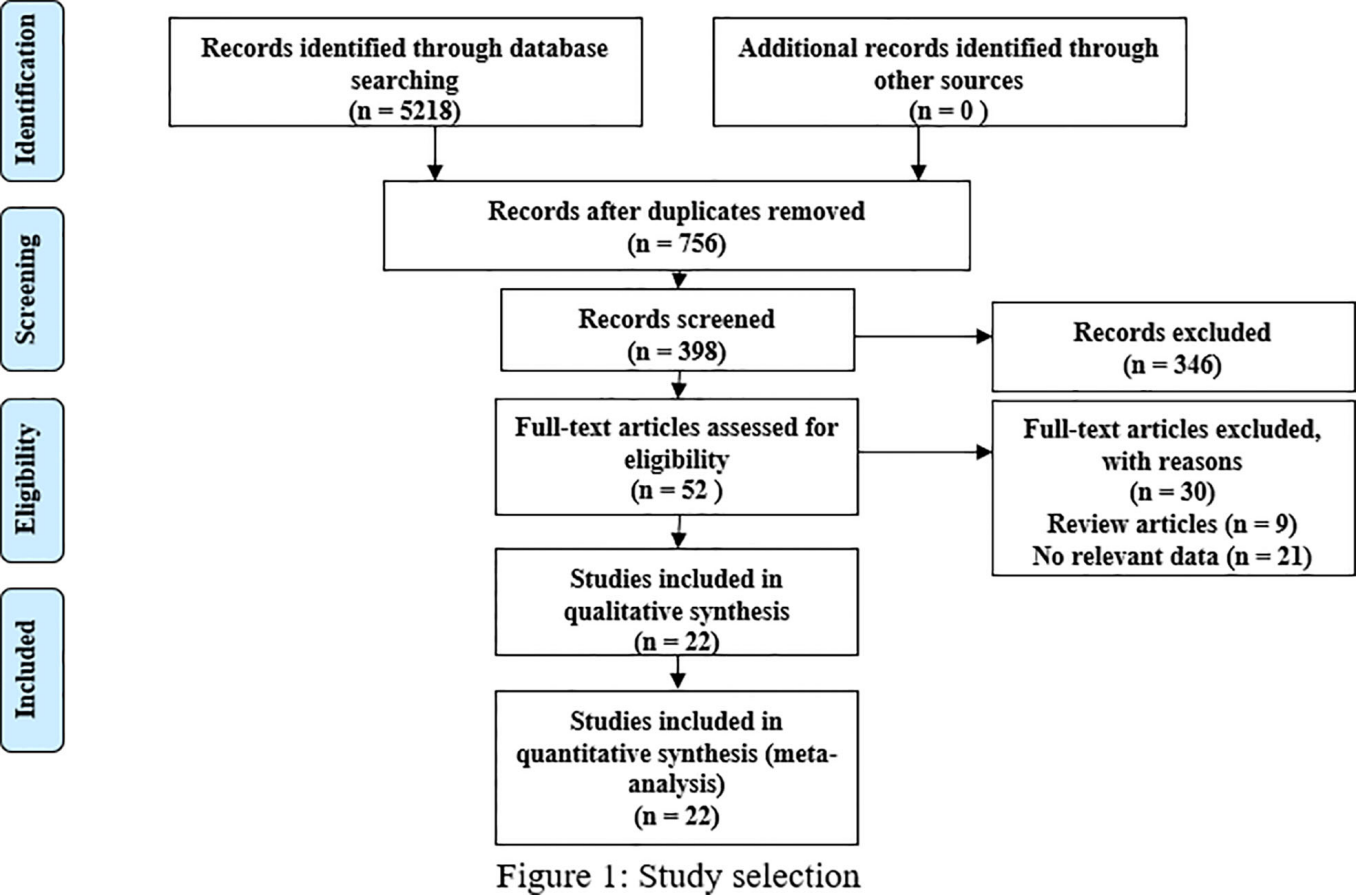
Flow of study selection.

Following the Preferred Reporting Items for Systematic Reviews and Meta-Analysis (PRISMA) guidelines, we included 22 studies that involved 3369 patients and satisfied our inclusion/exclusion criteria for meta-analysis. The preliminary literature search resulted in 5218 articles, and 756 studies remained after exclusion due to duplication. After scanning the titles and abstracts, we obtained 52 studies by excluding an additional 346 studies. After reading the full texts and review articles, we further excluded 30 studies that did not report sex hormone levels. Finally, 22 studies (14–30) (19, 31–33) were included in our analysis. The process of study identification and selection is shown in **Figure 1**.

### Description of included studies

In total, there were 3369 patients: 8 studies from Turkey (930 patients), 7 from Italy (975 patients), 5 from China (773 patients), and 1 each from France (118 patients), and the USA (152 patients). The patient ages ranged from 18 to 73 years old. Overall, there were nine cohort studies, six case–control studies, and seven cross-sectional studies. Virtually all respondents were male, with only four studies including female respondents. The articles were published during the period from 2020 to 2021. According to the NOS scores, all studies were deemed to be of high quality. The characteristics of the 22 eligible studies and their NOS scores are summarized in **Table 1**.

**Table 1:** Studies included in the meta-analysis.

Number	Author	Year	Country	Type of study	Sample size	Female/Male	Age (Mean±SD)	BMI (Mean±SD)	Sex hormone	NOS	Short description
**1**	**M. Infante**	**2021**	**Italy**	**Cross-sectional**	**59**	**Only Male**	**Survivors:64.10 ± 13.02 Non-survivors:68.23 ± 12.74**	**Survivors: 28.03 ± 1.95 Non-survivors: 28.5 ± 3.0**	**TT, E2, E2/T, Progesterone, PRL**	**8**	**The study aimed to test the correlation between serum levels of sex hormones [total testosterone, estradiol (E2), estradiol to testosterone (E2/T) ratio, progesterone), prolactin and 25-hydroxyvitamin D [25(OH)D]and markers of inflammation, coagulation and sepsis at admission in hospitalized men with COVID-19.**
**2**	**Abdullah Gul**	**2021**	**Turkey**	**Cross-sectional**	**29**	**Only Male**	**COVID-19 patients:31.21±5.48**	**COVID-19 patients: 27.05±2.34**	**FSH, LH, TT, PRL**	**8**	**The aim of the study has been to investigate the long-term effects of SARS-CoV-2 infection (COVID-19) and its relative treatment on male reproductive health.**
**3**	**Erdem Koç**	**2021**	**Turkey**	**Cross-sectional**	**21**	**Only Male**	**COVID-19 patients:32±6.30**	**COVID-19 patients: 25.62±2.12**	**FSH, LH, TT**	**7**	**The study aimed to evaluate the effect of COVID-19 on the semen parameters and sex-related hormone levels in infertile men.**
**4**	**Ling Ma**	**2021**	**China**	**Case-control**	**392**	**Only Male**	**patients with COVID-19:39.33±7.74 Control: 38.66±6.02**	**-**	**TT, FSH, LH, T/LH, FSH/LH**	**9**	**It’s the first report about semen assessment and sexhormone evaluation in reproductive-aged male COVID-19 patients.**
**5**	**Mustafa Zafer Temiz**	**2020**	**Turkey**	**Cross-sectional**	**30**	**Only Male**	**Control:36.64±9.63 COVID-19 patients Before treatment:38.00±8.28 COVID-19 patients After treatment:37.00±8.69**	**Control: 26.57 ± 2.71 COVID-19 patients Before treatment:25.55 ± 2.08 COVID-19 patients After treatment :26.55 ± 1.14**	**FSH, LH, PRL, TT, T/LH, FSH/LH, PRL/T**	**8**	**The study investigated whether there is a male reproductive system coronavirus disease-2019 (COVID-19) phenomenon.**
**6**	**Hui Xu**	**2020**	**China**	**Cohort**	**61**	**Only Male**	**COVID-19 patients: 57.33±14.62Control: 60.91±13.27**	**COVID-19 patients: 25.1±2.8 Control: 26.9±3.6**	**TT, FT, FSH, LH, PRL, E2, T/LH**	**7**	**The study aimed to assess whether SARS-CoV-2 infection can affect sex-related hormones and testicular function in recovering patients. In males infected with SARS-CoV-2, most sex-related hormones (T, FSH and** **LH levels) remain within the normal reference ranges after recovery from COVID-19, and no significant associations were observed between T level and disease duration or severity .**
**7**	**Marta Camici**	**2021**	**Italy**	**Case-control**	**48**	**Only Male**	**COVID-19 patients: 50.66±12.60 Control:50.33±11.03**	**-**	**TT, SHBG**	**8**	**The study aimed to investigate the association between sex hormones and the severity of coronavirus disease 2019 (COVID-19). A low level of testosterone was found to be a marker of clinical severity of COVID-19.**
**8**	**Andrea Salonia**	**2021**	**Italy**	**Case-control**	**567**	**Only Male**	**COVID-19 patients:57.66±12.66 Healthy Control:44.33±12.66**	**COVID-19 patients:27.9±4.24 Healthy Control:25.4±1.93**	**FSH, LH, TT, E2**	**9**	**The study aimed to assess: (a) circulating sex steroids levels in a cohort of 286 symptomatic men with laboratory- confirmed COVID- 19 at hospital admission compared to a cohort of 281 healthy men; and (b) the association between serum testosterone levels (tT), COVID- 19, and clinical outcomes.**
**9**	**Sezgin Okçelik**	**2020**	**Turkey**	**Case-control**	**44**	**Only Male**	**from 18 to 50 years**	**-**	**FSH, LH, TT**	**7**	**The study aimed to evaluate the testicular damage caused by COVID-19, Testosterone levels seem to decrease during acute COVID-19 infection, especially in the patient group with viral pneumonia.**
**10**	**Andrea Salonia**	**2021**	**Italy**	**Cohort**	**121**	**Only Male**	**COVID-19 patients: 57.00±12.00**	**-**	**FSH, FSH, TT, E2**	**8**	**The study aimed to assess total testosterone levels and the prevalence of total testosterone still suggesting for hypogonadism at 7-month follow-up in a cohort of 121 men who recovered from laboratory-confirmed COVID-19.**
**11**	**Giulia Rastrelli**	**2020**	**Italy**	**Cohort**	**31**	**Only Male**	**Transferred to IM :61.50±9.14 In charge in RICU:62.83±48.15 Transferred to ICU/deceased :73.00±27.84**	**-**	**TT, SHBG, LH**	**7**	**The study aimed to estimate the association between T level and SARS-CoV-2 clinical outcomes (defined as conditions requiring transfer to higher or lower intensity of care or death) in a cohort of patients admitted in the respiratory intensive care unit (RICU).**
**12**	**Selahittin Çayan**	**2020**	**Turkey**	**Cohort**	**221**	**Only Male**	**Asymptomatic group:34.83 ± 12.51 IMU group:44.54 ± 17.63 ICU group:56.8 ± 18.57**	**Asymptomatic group:23.87±3.6 IMU group:23.62±3.6 ICU group:24.18±2.83**	**FSH, LH, TT, Prolactin, E2**	**8**	**The study aimed to investigate effect of serum total testosterone and its relationship with other laboratory parameters on the prognosis of coronavirus disease 2019 (COVID-19) in severe acute respiratory syndrome coronavirus 2 (SARS-CoV-2) infected male patients.**
**13**	**Sandeep Dhindsa**	**2021**	**USA**	**Cohort**	**152**	**COVID-19 patients :152 (62/90)**	**Severe COVID-19: Men :68 ± 11; Women:68±14 Without Severe COVID-19: Men: 55 ± 15; Women:51±19**	**Severe COVID-19: Men: 26.7±6.0; Women:32.6±9.3 Without severe COVID-19: Men: 30.0±8.0 ; Women:34.3±8.1**	**TT, E2, E2/T**	**8**	**The study aimed to investigate the association of concentrations of serum testosterone, estradiol, and insulinlike growth factor 1 (IGF-1, concentrations of which are regulated by sex hormone signaling) with COVID-19 severity.**
**14**	**Anna Beltrame**	**2022**	**Italy**	**Cohort**	**120**	**COVID-19 patients :120 (52/68)**	**50 years and over, stratified by sex and outcome.**	**-**	**TT, E2, Progesterone**	**7**	**The study investigated sex hormone levels and their association with outcomes in COVID-19 patients, stratified by sex and age.In males, higher testosterone seems to be protective against any considered outcome.**
**15**	**Ting Ding**	**2020**	**China**	**Cross-sectional**	**78**	**Only Female**	**younger than 60 years of age**	**-**	**E2, AMH, LH, TT, FSH, FSH/LH、PRL**	**7**	**The study aimed to find the factors that potentially protect females from COVID-19, Menopause is an independent risk factor for female COVID-19 patients.**
**16**	**Ahmet Emre Cinislioglu**	**2021**	**Turkey**	**Cohort**	**450**	**Only Male**	**COVID-19 patients:64.9±11.6 Control:67.2±13.6**	**COVID-19 patients: 25.9±3.8 Control:26.4±3.1**	**TT, FSH, LH, T/L**	**8**	**The study aimed to investigate the relationship of serum testosterone with other laboratory parameters on the prognosis of coronavirus disease-19 (COVID-19) in male patients with COVID-19 diagnosis.**
**17**	**Shufa Zheng**	**2021**	**China**	**Cross-sectional**	**61**	**Only Male**	**Non-ICU:50.03±18.87 ICU:63.26±17.54**	**-**	**TT**	**8**	**The purpose of this study is to analyze the relationship between testosterone changes and disease severity in male patients with COVID-19 and to compare the differences in transcriptome expression in patients with different testosterone levels.**
**18**	**Emre Urhan**	**2021**	**Turkey**	**case-control**	**54**	**COVID-19 patients : 43 (19/24) Control : 11 (5/6)**	**COVID-19 patients:44.28±10.76 Control:44.18±12.41**	**COVID-19 patients:31.04±5.92 Control:29.92±3.29**	**PRL**	**8**	**The study investigated the pituitary functions three to seven months after acute COVID-19 infection.**
**19**	**Stefano Salciccia**	**2020**	**Italy**	**Cross-sectional**	**29**	**Only Male**	**None O2 assistance:57.00±58.60 Invasive O2 assistance:63.66±40.69**	**-**	**TT**	**7**	**The study aimed to evaluate whether serum TT levels among a cohort of 29 COVID-19 men at the time of hospital admission were associated with the need for “invasive” oxygenation strategy.** **and may allow for patient monitoring and predict disease** **outcome.**
**20**	**Tugce Apaydin**	**2022**	**Turkey**	**Cohort**	**81**	**Only Male**	**Mild-moderate COVID-19 patients:43.16±28.94 Severe COVID-19 patients:46.33±25.82**	**-**	**FSH, LH, TT, FT, SHBG**	**8**	**The study aimed to evaluate the acute and chronic effects of coronavirus disease 2019 on gonadal functions.**
**21**	**Ling Ma**	**2020**	**China**	**Case-control**	**181**	**Only Male**	**Men with COVID-19: 38.33±6.03 Age-matched healthy men:38.00±4.51**	**-**	**TT, FSH, LH, PRL, T/LH, FSH/LH, AMH, E2, T/E2**	**9**	**This study provides the first direct evidence about the influence of medical condition of COVID-19 on male sex hormones, alerting more attention to gonadal function evaluation among patients recovered from SARS-CoV-2 infection, especially the reproductive-aged men.**
**22**	**Kamila Kolanska**	**2021**	**France**	**Cohort**	**118**	**Only Female**	**COVID-19 positive:35.7±4.2 COVID-19 negative:34.5±4.5**	**COVID-19 positive:23.1±3.7COVID-19 negative:24.3±5.5**	**AMH**	**9**	**The aim of this prospective study was to evaluate the effect of mild COVID-19 infection on the ovarian reserve of women undergoing an assisted reproductive technology (ART) protocol.**

## Overall analysis

### Results of the meta-analysis of free testosterone levels in the COVID-19 group and controls

**Figure 2:**
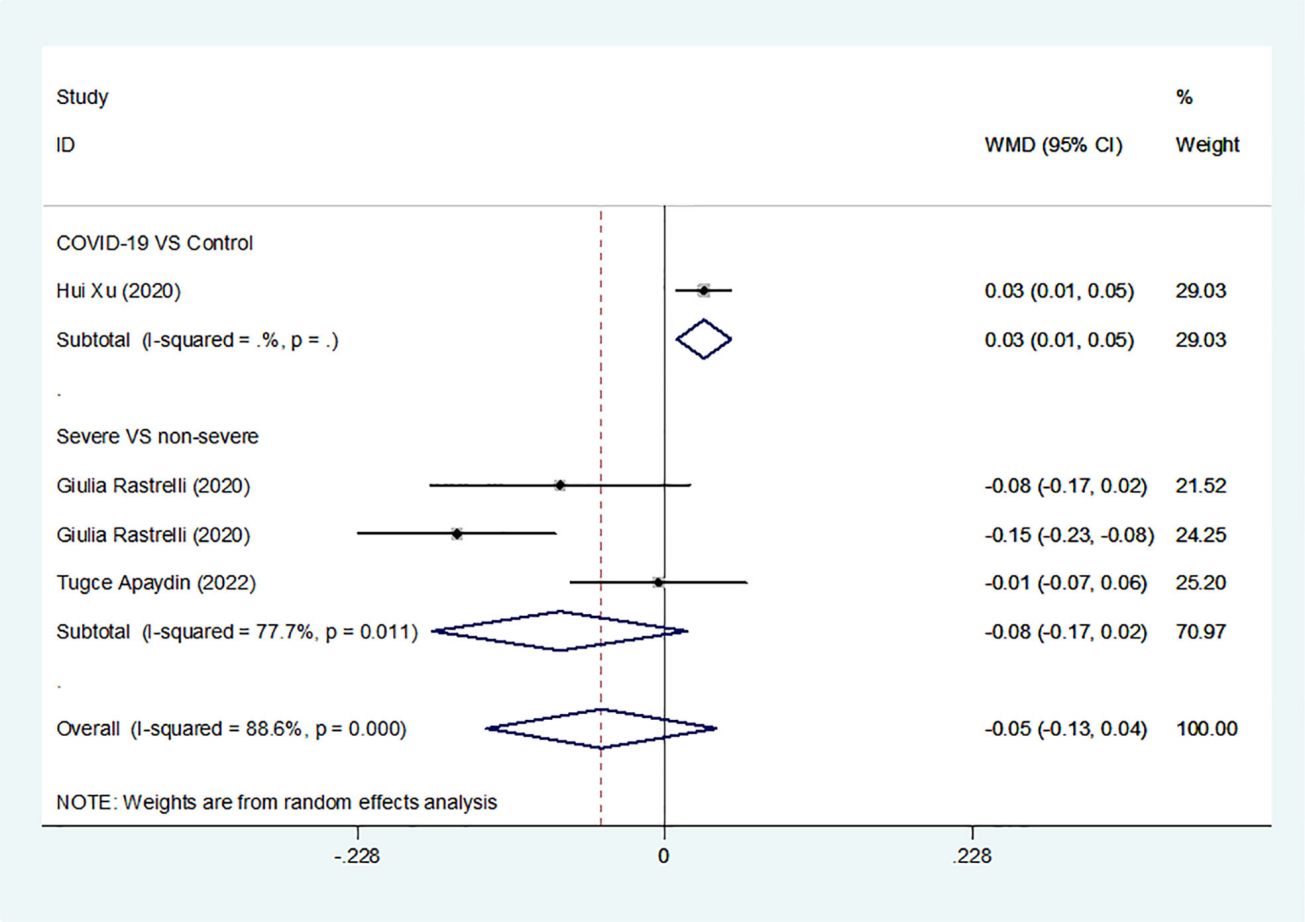
FT levels in people with COVID-19 versus those without COVID-19.

Overall, we found a milieu of clinical androgenicity (sexual function, bone density and haematopoiesis) resulting from the interaction between polyQ polymorphism in the androgen receptor (AR) and serum testosterone levels. In particular, the calculated FT correlated better than total testosterone (TT) with all relevant clinical parameters of androgenicity, and androgenicity was reduced when the AR showed a large number of CAG repeats. We identified 3 studies published from 2020 to 2022 (three cohort studies) that presented results on FT levels and COVID-19, including a total of 194 subjects. Subgroup analyses were stratified by disease severity (patients with COVID-19 vs. controls; patients with severe COVID-19 vs. those without severe COVID-19. In 1 study that measured levels of FT in patients with COVID-19 or controls, the aggregated WMD was 0.03, with a 95% CI of 0.01 and 0.05. Based on 3 studies that compared patients with severe COVID-19 vs. those without severe COVID-19, the pooled WMD was -0.08 (95% CI: -0.17 to 0.02) (**Table 2** and **Figure 2**). Egger’s test showed that no publication bias existed among the included studies (*p* > 0.05). The publication bias analysis showed that the funnel plot was nearly symmetrical (**Figure 7A**). The results of the sensitivity analyses indicated that the conclusions were robust (**Figure 8A**). The above results revealed that patients with COVID-19 have no significance FT levels than subjects without COVID-19.

**Table 2:** Sex hormone levels.

Sex hormone parameters	Subgroup	Study	Number of patients	WMD	95% CI	I2
**FT**	**COVID-19 VS Control**	**1**	**61**	**0.03**	**(0.01, 0.05)**	**0.00%**
	**Severe VS non-severe**	**3**	**133**	**-0.08**	**(-0.17, 0.02)**	**70.97%**
**FSH**	**Cohort**	**4**	**1249**	**2.05**	**(1.03, 3.07)**	**72.30%**
	**Case-control**	**3**	**838**	**0.02**	**(-0.3, 0.34)**	**0.00%**
	**Cross-sectional**	**3**	**178**	**0.22**	**(-0.93, 1.38)**	**41.30%**
	**Italy**	**2**	**809**	**-1.13**	**(-3.15, 0.88)**	**93.20%**
	**Turkey**	**6**	**1658**	**1.21**	**(0.24, 2.19)**	**87.70%**
	**China**	**4**	**790**	**-0.08**	**(-0.85, 0.69)**	**45.50%**
	**Male**	**11**	**3409**	**0.58**	**(-0.16,1.32)**	**89.50%**
	**Female**	**1**	**78**	**19.65**	**(-1.12,40.42)**	**0.00%**
**LH**	**COVID-19 VS Control**	**7**	**1737**	**1.42**	**(0.21, 2.63)**	**95.30%**
	**Severe VS non-severe**	**6**	**1031**	**-0.48**	**(-0.59, 0.63)**	**64.50%**
	**Cohort**	**5**	**880**	**1.11**	**(0.06, 2.16)**	**84.20%**
	**Case-control**	**4**	**1230**	**1.57**	**(0.33, 2.82)**	**93.70%**
	**Cross-sectional**	**4**	**209**	**-0.17**	**(-1.42, 1.08)**	**51.70%**
	**Younger than 50 years old**	**6**	**841**	**1.28**	**(0.06, 2.5)**	**90.90%**
	**Old than 50 years old**	**7**	**2447**	**0.66**	**(-0.43, 1.75)**	**94.40%**
	**Male**	**12**	**3461**	**0.9**	**(0.10, 1.71)**	**93.70%**
	**Female**	**1**	**78**	**11.3**	**(-7.88, 30.48)**	**0.00%**
**PRL**	**Cohort**	**2**	**360**	**1.13**	**(0.16, 2.10)**	**0.00%**
	**Case-control**	**3**	**294**	**2.26**	**(-13.60, 18.13)**	**99.20%**
	**Cross-sectional**	**3**	**156**	**0.12**	**(-0.91, 1.15)**	**0.70%**
	**Male**	**6**	**739**	**0.68**	**(-0.01,1.37)**	**0.00%**
	**Female**	**1**	**78**	**-0.77**	**(-5.28,3.74)**	**0.00%**
**E2**	**COVID-19 VS Control**	**4**	**857**	**6.49**	**(0.27, 12.7)**	**90.30%**
	**Severe VS non-severe**	**5**	**548**	**0.51**	**(-3.22, 4.25)**	**61.90%**
	**Non-survivor VS survivor**	**2**	**179**	**7.36**	**(-7.98, 22.69)**	**85.90%**
	**Male**	**9**	**1729**	**0.15**	**(-0.16,0.46)**	**87.60%**
	**Female**	**2**	**182**	**-5.36**	**(-32.44,21.71)**	**79.60%**
**E2/T**	**COVID-19 VS Control / Severe VS non-severe**	**3**	**330**	**0.89**	**(-2.17, 3.96)**	**76.10%**
**Progesterone**	**Severe VS non-severe**	**3**	**377**	**0.01**	**(-0.05, 0.07)**	**73.40%**
	**Male**	**2**	**195**	**0.24**	**(-0.48,0.97)**	**76.10%**
	**Female**	**2**	**182**	**-0.02**	**(-0.98,0.42)**	**75.20%**
**SHBG**	**COVID-19 VS Control / Severe VS non-severe**	**3**	**160**	**-8.69**	**(-17.20, -0.18)**	**0%**
**T/LH**	**COVID-19 VS Control / Severe VS non-severe**	**4**	**1778**	**-1.1**	**(-1.42, -0.79)**	**85.30%**
**FSH/LH**	**COVID-19 VS Control / Severe VS non-severe**	**3**	**651**	**-0.66**	**(-0.74, -0.57)**	**0.00%**

### Results of the meta-analysis of FSH levels in the COVID-19 group and controls

**Figure 3:**
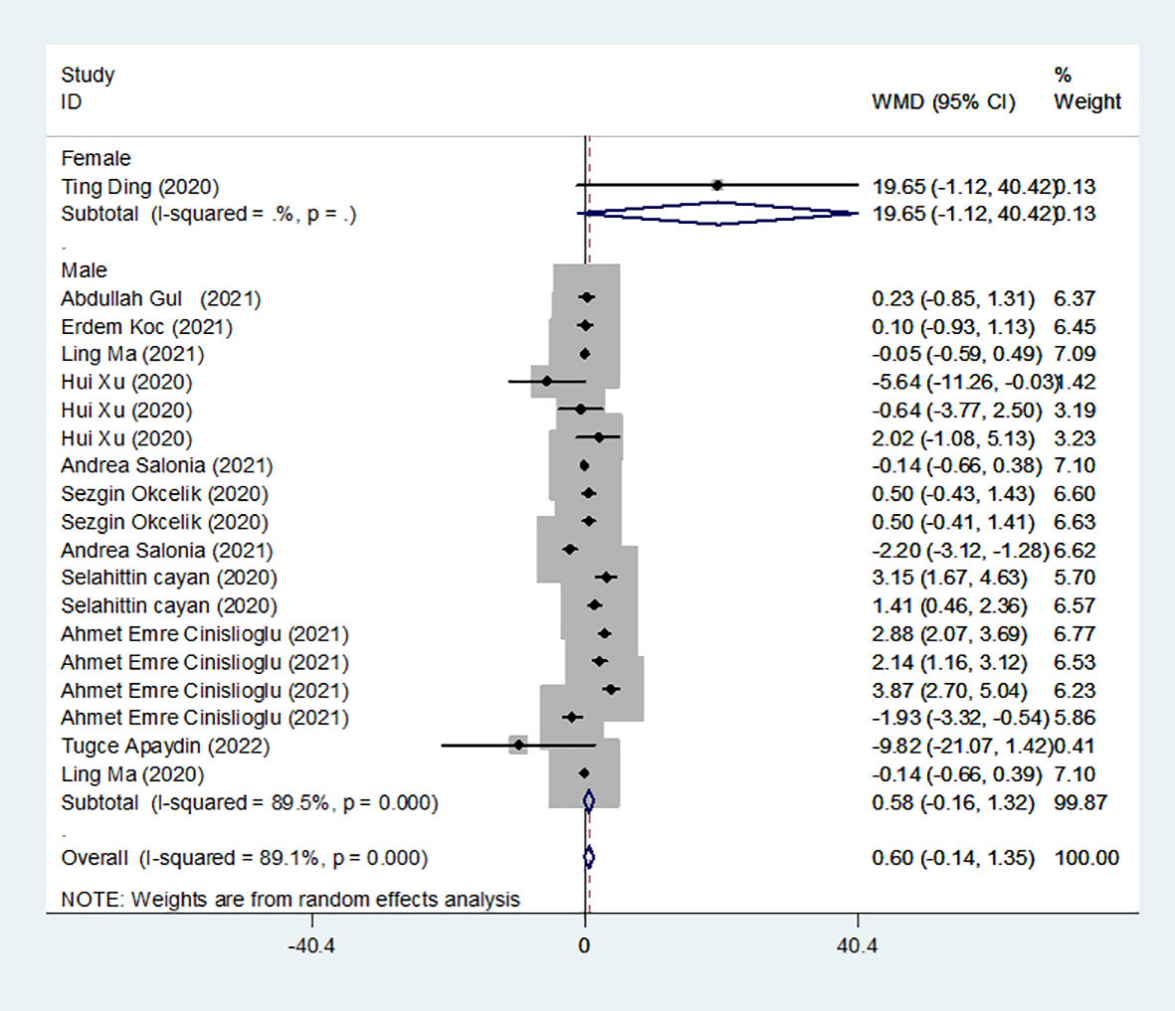
FSH levels in people with COVID-19 versus those without COVID-19.

We identified 12 studies published from 2020 to 2021 (four case–control studies, five cohort studies, and three cross-sectional studies) that presented results on FSH levels and COVID-19. The 12 studies included a total of 3257 subjects. The effect size from the random-effects model showed no significant changes in FSH levels (pooled WMD: 0.60, CI: -0.14 and 1.35) (**Figure 3**). The heterogeneity test results found obvious heterogeneity (*I^2^* = 89.1%, *p* = 0.000). Sensitivity analysis confirmed that no individual study influenced the overall results.

Subgroup analyses were stratified by sex (male, female), study design (cohort, case–control and cross-sectional) and country (Italy, Turkey and China). In 1 study with females that assessed levels of FSH in patients, the aggregated WMD was 19.65, with a 95% CI of -1.12 and 40.42. From 11 studies with males, the pooled WMD was found to be 0.58 (95% CI: -0.16 to 1.32). In 4 studies with a cohort design that assessed the levels of FSH in patients, the aggregated WMD was 2.05 with a 95% CI of 1.03 and 3.07. From 3 studies using a case–control design, the pooled SDM was found to be 0.02 (95% CI: -0.30 to 0.34). From 3 studies with a cross-sectional design, the pooled WMD was found to be 0.22 (95% CI: -0.93 to 1.38). Subgroup analysis was performed according to country, and decreased FSH levels were found in Turkey but not in Italy or China (pooled WMD: 1.21, CI: 0.24 and 2.19 for Turkey; pooled WMD: -1.13, CI: -3.15 and 0.88 for Italy; pooled WMD: -0.08, CI: -0.85 and 0.69 for China) (**Table 2**). Egger’s test showed that no publication bias was present among the included studies (*p* > 0.05). The publication bias analysis showed that the funnel plot was nearly symmetrical (**Figure 7B**). The results of the sensitivity analyses indicated that the conclusions were robust (**Figure 8B**). The above results revealed that patients with COVID-19 have higher FSH levels than individuals without COVID-19.

### Results of the meta-analysis of LH levels in the COVID-19 group and controls

**Figure 4:**
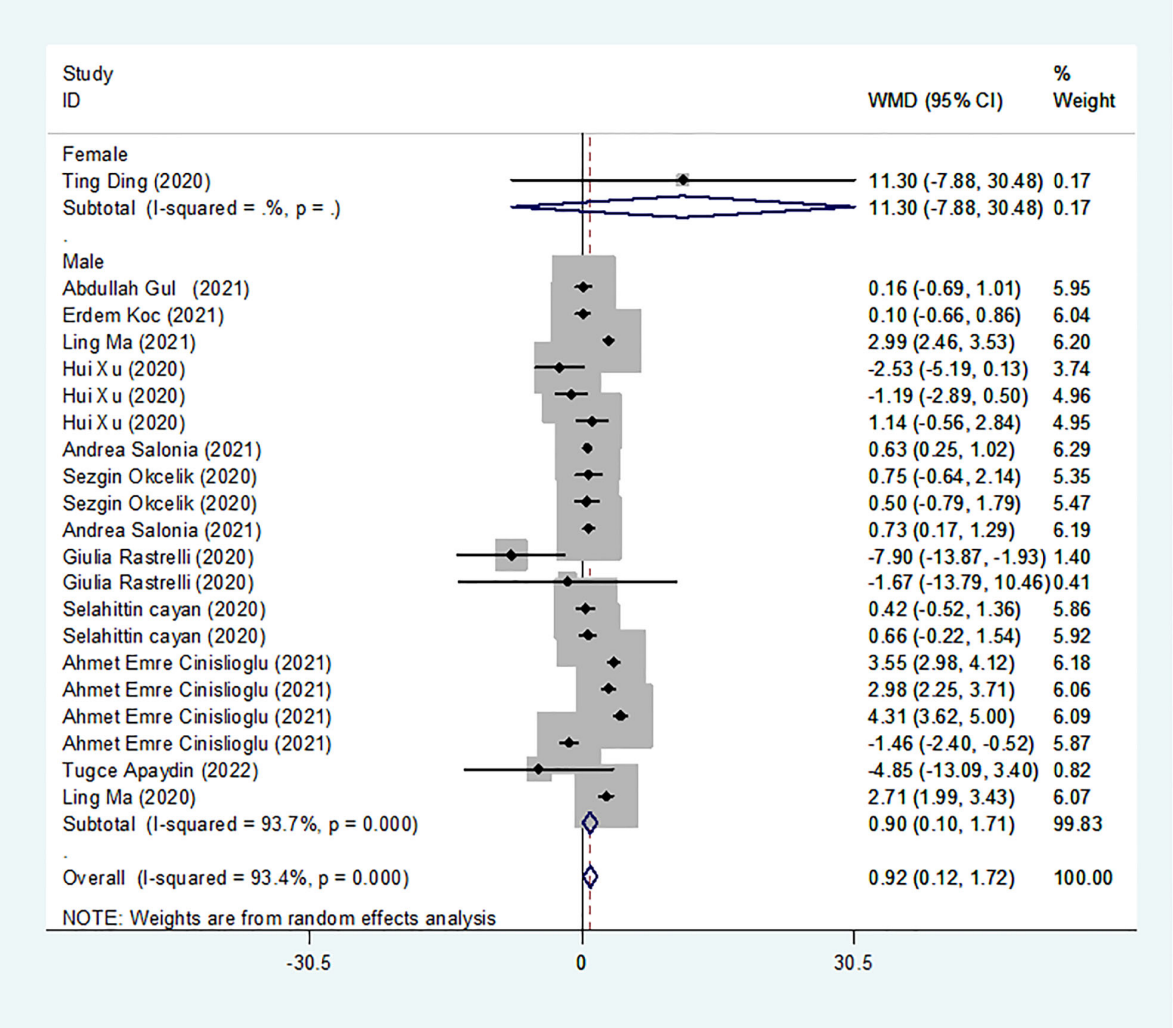
LH levels in people with COVID-19 vsersus those without COVID-19.

We identified 13 studies published from 2020 to 2021 (four case–control studies, five cohort studies, and four cross-sectional studies) that presented results on LH levels and COVID-19. The 13 studies included a total of 3288 subjects. The effect size from the random-effects model showed a significant increase in LH levels (pooled WMD: 0.92, CI: 0.12 and 1.72) (**Figure 4**). The heterogeneity test results found obvious heterogeneity (*I^2^* = 93.4%, *p* = 0.000). Sensitivity analysis confirmed that no individual study influenced the overall results.

Subgroup analyses were stratified by sex (male, female), disease severity (patients with COVID-19 vs. controls; patients with severe COVID-19 vs. those without severe COVID-19), age (younger than 50 years old vs. older than 50 years old), and study design (cohort, case–control and cross-sectional). In 1 study that measured levels of LH in female patients with COVID-19 or controls, the aggregated WMD was 11.30, with a CI of -7.88 and 30.48. From 12 studies that compared male patients with COVID-19 or those without COVID-19, the pooled WMD was 0.90 (95% CI: 0.10 to 1.71). In 7 studies that measured the levels of LH in patients with COVID-19 or controls, the aggregated WMD was 1.42 with a CI of 0.21 and 2.63. From 6 studies that compared patients with severe COVID-19 with those without severe COVID-19, the pooled WMD was found to be -0.48 (95% CI: -1.59 to 0.63). In 5 studies with a cohort design that assessed the levels of LH in patients, the aggregated WMD was 1.11 with a CI of 0.06 and 2.16. From 4 studies with a case–control design, the pooled WMD was found to be 1.57 (95% CI: 0.33 to 2.82). From 4 studies that used a cross-sectional design, the pooled SDM was found to be -0.17 (95% CI: -0.93 to 1.38). In a subgroup analysis according to age, LH levels were found to be significantly higher only in patients younger than 50 years old but not in those older than 50 years old (pooled WMD: 1.28, CI: 0.06 and 2.5 for younger than 50 years old; pooled WMD: 0.66, CI: -0.43 and 1.75 for older than 50 years old) (**Table 2**). Egger’s test showed that no publication bias was present among the included studies (*p* > 0.05). The publication bias analysis showed that the funnel plot was nearly symmetrical (**Figure 7C**). The results of the sensitivity analyses indicated that the conclusions were robust (**Figure 8C**). The above results revealed that patients with COVID-19 present higher LH levels than subjects without COVID-19.

### Results of the meta-analysis of PRL levels in COVID-19 and control groups

**Figure 5:**
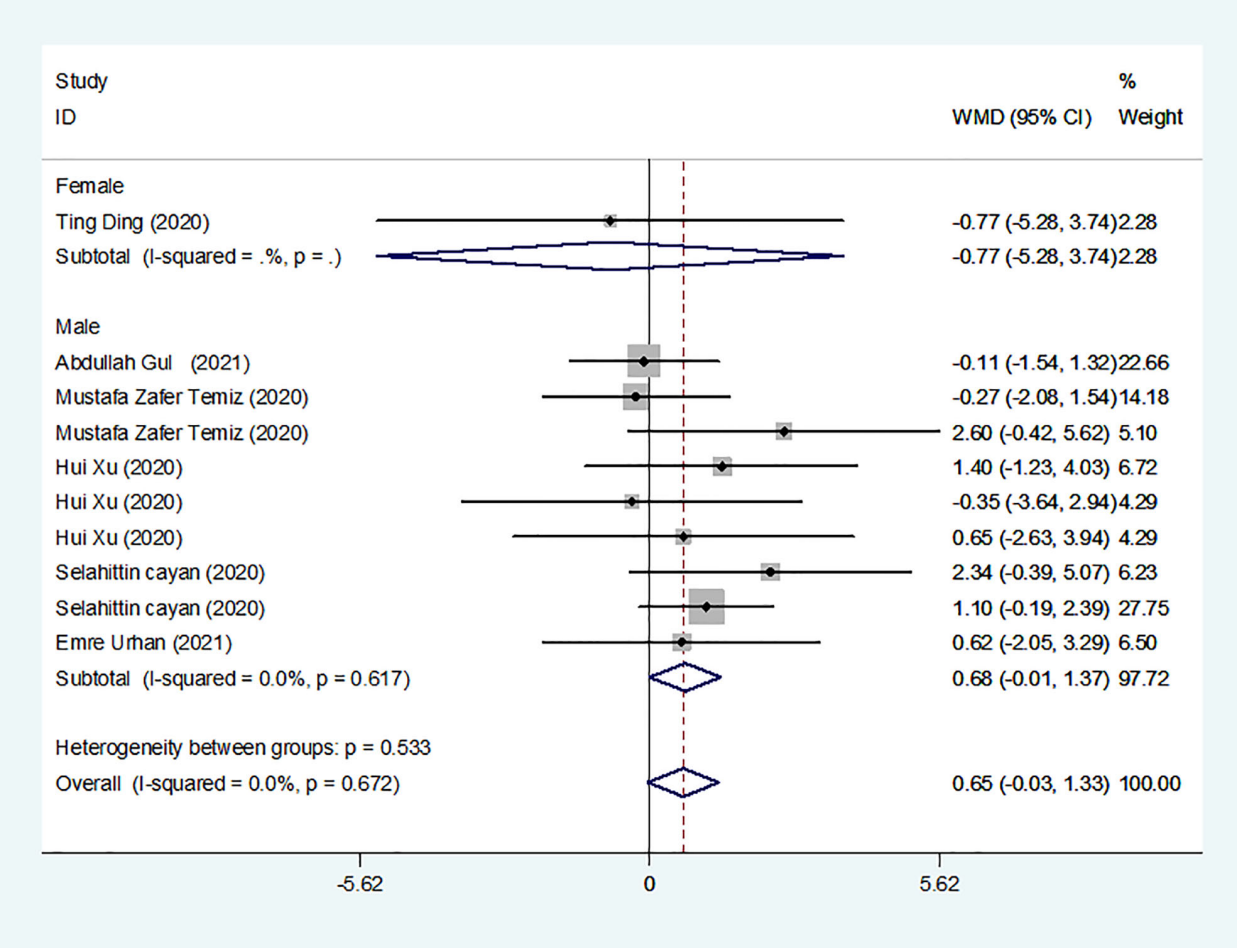
PRL levels in people with COVID-19 versus those without COVID-19.

We identified 8 studies published from 2020 to 2021 (three case–control studies, two cohort studies, and three cross-sectional studies) that presented results on PRL levels and COVID-19. The 8 studies included a total of 810 subjects. The effect size from the fixed-effects model showed a significant increase in PRL levels (pooled WMD: 0.65, CI: -0.03 and 1.33) (**Figure 5**).

Subgroup analyses were stratified by sex (male, female), study design (cohort, case–control and cross-sectional). In 1 study with females that detected levels of PRL in patients, the aggregated WMD was -0.77, with a CI of -5.28 and 3.74. From 6 studies involving males, the pooled WMD was 0.68 (95% CI: -0.01 to 1.37). In 2 studies with a cohort design that detected the levels of PRL in patients, the aggregated WMD was 1.13 with a CI of 0.16 and 2.10. From 3 studies with a case–control design, the pooled WMD was found to be 2.26 (95% CI: -13.60 to 18.13). From 3 studies that used a cross-sectional design, the pooled WMD was found to be 0.12 (95% CI: -0.91 to 1.15). Egger’s test showed that no publication bias existed among the included studies (*p* > 0.05). The publication bias analysis showed that the funnel plot was nearly symmetrical (**Figure 7D**). The results of the sensitivity analyses indicated that the conclusions were robust (**Figure 8D**). The above results revealed that patients with COVID-19 present no significant changes in PRL levels compared to subjects without COVID-19.

### Results of the meta-analysis of E_2_ levels in the COVID-19 group and controls

**Figure 6:**
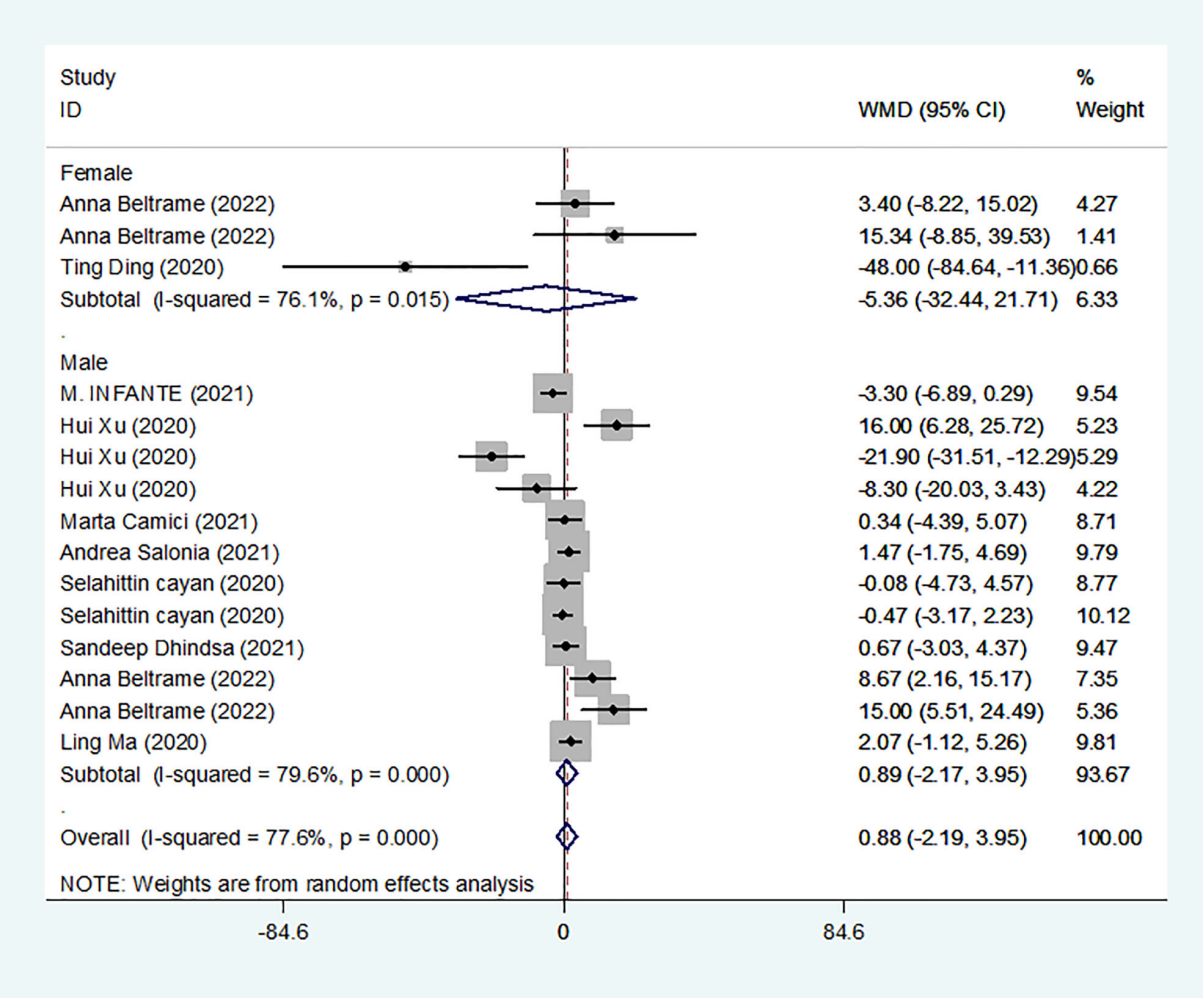
E_2_ levels in people with COVID-19 versus those without COVID-19.

We identified 10 studies published from 2020 to 2021 (three case–control studies, four cohort studies, and two cross-sectional studies) that presented results on E_2_ levels and COVID-19. The 10 studies included a total of 1584 subjects. The effect size from the random-effects model showed no significant difference in PRL levels (pooled WMD: 0.88, CI: -2.19 and 3.95) (**Figure 6**). The heterogeneity test results found obvious heterogeneity (*I^2^* = 77.6%, *p* = 0.000).

**Figure 7:**
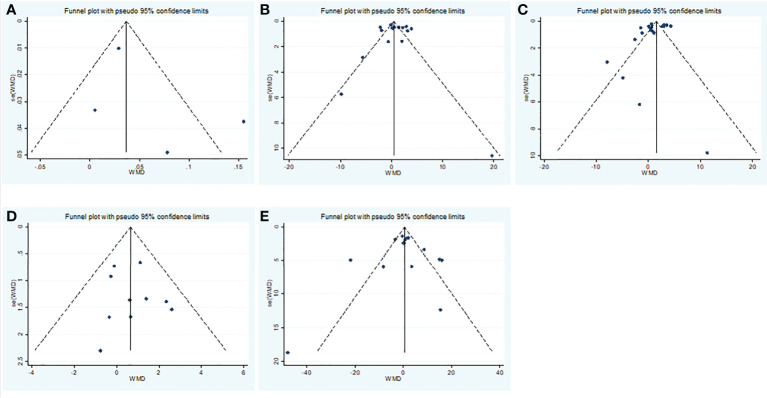
Publication bias funnel plots of the WMD for **(A)** TT, **(B)** FSH, **(C)** LH, **(D)** PRL, **(E)** E_2_ and COVID-19.

**Figure 8:**
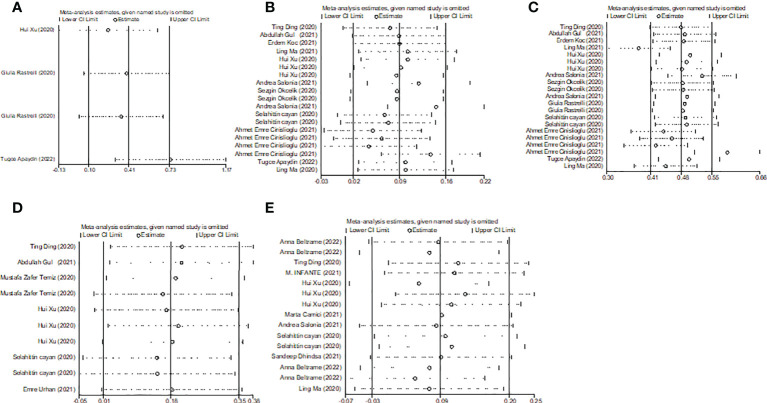
Sensitivity analyses of the WMD for **(A)** TT, **(B)** FSH, **(C)** LH, **(D)** PRL, **(E)** E_2_ and COVID-19.

Subgroup analyses were stratified by sex (male, female), disease severity (patients with COVID-19 vs. controls; patients with severe COVID-19 vs. those without severe COVID-19; non-survivors vs. survivors). In 2 studies that measured levels of E_2_ in female patients with COVID-19 or controls, the aggregated WMD was -5.36, with a CI of -32.44 and 21.71. Based on 7 studies comparing male patients with and without COVID-19, the pooled WMD was found to be 0.89 (95% CI: -2.17 to 3.95). In 4 studies that measured the levels of E_2_ in patients with COVID-19 or controls, the aggregated WMD was 6.49 with a CI of 0.27 and 12.7. From 5 studies that compared patients with severe COVID-19 with those without severe COVID-19, the pooled WMD was found to be 0.51 (95% CI: -3.22 to 4.25). Non-survivors vs. survivors were assessed in only 2 studies, with a WMD value of 7.36 (95% CI: -7.98 to 22.69). Egger’s test showed that no publication bias existed among the included studies (*p* > 0.05). The publication bias analysis showed that the funnel plot was nearly symmetrical (**Figure 7E**). The results of the sensitivity analyses indicated that the conclusions were robust (**Figure 8E**). The above results revealed that patients with COVID-19 present lower E_2_ levels than subjects without COVID-19.

### Results of the meta-analysis of progesterone, SHBG, T/LH, FSH/LH, and E_2_/T levels in the COVID-19 group and controls

We identified 3 studies published from 2020 to 2021 that presented results on progesterone levels and COVID-19. The 3 studies included a total of 196 subjects and 181 COVID-19 patients. The effect size from the random-effects model showed a significant increase in progesterone levels (pooled WMD: 0.01, CI: -0.05 and 0.07) (**Table 2**). The heterogeneity test results found obvious heterogeneity (*I^2^* = 73.4%, p = 0.000).

We identified 3 studies published from 2020 to 2021 that presented results on SHBG levels and COVID-19. The 3 studies included a total of 120 subjects and 61 COVID-19 patients. The effect size from the random-effects model showed a significant increase in SHBG levels (pooled WMD: -8.69, CI: -17.20 and -0.18) (**Table 2**). The heterogeneity test results found obvious heterogeneity (*I^2^* = 0%, *p* = 0.000).

We identified 4 studies published from 2020 to 2021 that presented results on T/LH levels and COVID-19. The 4 studies included a total of 874 subjects and 1088 COVID-19 patients. The effect size from the random-effects model showed a significant increase in T/LH values (pooled WMD: -0.95, CI: -1.36 and -0.55) (data not shown). The heterogeneity test results found obvious heterogeneity (*I^2^* = 93.4%, p = 0.000). Sensitivity analyses were performed and showed that the study type was the main factor impacting the results (pooled WMD: -1.10, CI: -1.42 and -0.79) (**Table 2**).

We identified 3 studies published from 2020 to 2021 that presented results on FSH/LH levels and COVID-19. The 3 studies included a total of 434 subjects and 217 COVID-19 patients. The effect size from the random-effects model showed a significant increase in FSH/LH values (pooled WMD: -0.66, CI: -0.74 and -0.57) (**Table 2**). The heterogeneity test results found no heterogeneity (*I^2^* = 0%, *p* = 0.000).

We identified 3 studies published from 2020 to 2021 that presented results on E_2_/T levels and COVID-19. The 3 studies included a total of 144 subjects and 186 COVID-19 patients. The effect size from the random-effects model showed a significant increase in E_2_/T values (pooled WMD: 0.40, CI: 0.18 and 0.63) (**Table 2**). The heterogeneity test results found no heterogeneity (*I^2^* = 26.4%, *p* = 0.000).

## Discussion

### Association between COVID-19 and sex hormone levels

To the best of our knowledge, our meta-analysis is the first to systematically assess the potential correlation between COVID-19 and sex hormone levels. In total, we included 22 studies for estimation of the effect size for sex hormone levels. Overall, patients with COVID-19 exhibited a significant change in the levels of sex hormones. Specifically, COVID-19 patients showed a significant decrease in the levels of T/LH, FSH/LH, and SHBG hormones. In contrast, COVID-19 patients displayed a significant increase in the levels of LH, and E_2_/T. There were no significant changes in levels of FT, FSH, PRL, E_2_, or progesterone. Sensitivity analysis and subgroup analyses both identified an obvious association between sex hormone levels and the risk of COVID-19.

Low SHBG levels seem to correlate with worse COVID-19 prognosis (**Table 2**). SHBG levels are known to be inversely related to obesity and insulin-resistance (34). Increasing evidence demonstrates that obesity is reversely associated with the development of COVID-19 (35). Thus, low plasma SHBG level predicts the severity of COVID-19.

Notably, this is closely linked to the infection-related failure of homeostatic HPG axis feedback to compensate for weakened AR signalling (36). Production of testosterone by testicular Leydig cells is tightly regulated by the hypothalamic-pituitary-gonadal (HPG) axis, forming a homeostatic negative feedback loop (37). Secondary hypogonadism involves pathology of the pituitary or hypothalamus, leading to disturbance in the HPG axis and subsequently in reduced testosterone (38). In addition, the overall milieu of clinical androgenicity (sexual function, bone density and haematopoiesis) results from interaction between polyQ polymorphism in the androgen receptor (AR) and serum testosterone (T) levels. The AR gene, located on the X chromosome, encodes a member of the receptor group that binds to and mediates the actions of androgens (39). AR gene polymorphisms and testosterone level may enhance the behaviours involved in obtaining and maintaining high social status and reproductive success in men (40). Therefore, HPG axis insufficiency and androgen receptor polymorphisms are jointly involved in testosterone function. According to the present meta-analysis, higher gonadotropin concentrations (LH) may increase COVID-19 risk and severity, whereas FT was relatively lower in these patients. These results suggest a decrease in peripheral organ function and a compensatory increase in central function. While, a compensatory increase in central function” which may be nonetheless insufficient due to the “infection-related failure of homeostatic HPG axis feedback.

Many risk factors associated with the progression of COVID-19 to a severe and critical stage have been identified, including old age, male sex, underlying comorbidities such as hypertension, diabetes, obesity, chronic lung disease, heart, liver and kidney diseases, and tumours, clinically apparent immunodeficiencies, local immunodeficiencies such as early type I interferon secretion capacity, and pregnancy. Sex hormones such as oestrogen and testosterone as well as sex chromosome complement likely contribute to sex differences in blood pressure (BP) and cardiovascular disease (CVD). At the cellular level, differences in cell senescence pathways may contribute to increased longevity in women and may also the limit organ damage caused by hypertension. In addition, many lifestyle and environmental factors - such as smoking, alcohol consumption and diet - may influence BP and CVD in a sex-specific manner. Evidence suggests that cardioprotection in women is lost under conditions of obesity and type 2 diabetes mellitus. Treatment strategies for hypertension and CVD that are tailored according to sex may lead to improved outcomes for affected patients. We also found that FSH and LH levels to be significantly increased in female COVID-19 patients. However, in male patients, only LH levels were significantly increased, and there was no significant difference in FSH expression. Treatment strategies for COVID-19 that are tailored according to sex may lead to improved outcomes for patients. This also reflects the different manifestations of sex in disease.

### Underlying mechanisms of COVID-19 effects on sex hormone levels

In COVID-19, SARS-CoV-2 may directly act on ACE2-positive spermatogonia, Sertoli cells and Leydig cells, resulting in disruption of spermatogenesis and male gonadal function (41).

Furthermore, in addition to the direct damage to the testes by viruses, other factors, such as fever, inflammation, and dysregulation of the hypothalamic-pituitary-gonadal axis (HPG axis), may also play a role in testosterone secretion or sperm production (42).

During viral infection, virus-induced inflammation leads to systemic or local production of cytokines, such as interleukin-6 (IL-6), tumor necrosis factor-α (TNF-α), interferon (IFN) and monocyte chemoattractant protein 1 (MCP-1). These cytokines are harmful to testicular cells. For example, IL-6 inhibits the differentiation of Leydig cells. In addition, IFN-γ has been shown to suppress the expression of the rate-limiting enzyme steroidogenic acute regulatory protein (StAR) to inhibit testosterone production (43–45). In addition, COVID-19 has also been reported within the central nervous system, including increased antidiuretic hormone secretion (46). The emotional, physical, or psychological stresses and pain associated with infections can affect the hypothalamohypophyseal axis (47). Thus, abnormalities in the hypothalamic-pituitary axis and abnormalities in LH secretion rhythm may also be a possible cause.

Aromatase is the enzyme responsible for the conversion of testosterone and androstenedione into E_2_ and oestrone, respectively. In men, aromatase is expressed in the testes (mainly in Leydig cells) as well as in a number of extragonadal sites, including adipose tissue, bone, the breasts and the brain (48). The upregulation of aromatase enzyme production in adipose tissue during critical illness (as a possible consequence of the excessive production of proinflammatory cytokines) may promote the conversion of testosterone to oestradiol (49–52).

Inflammatory mediators contribute to the suppression of the gonadal axis, and an increased metabolic clearance rate of testosterone may be the underlying cause of lower testosterone levels (53, 54).

There was no significant difference in the values of oestradiol in patients with COVID-19. The probable cause is that a potential upregulation of aromatase enzyme production in adipose tissue during COVID-19, possibly due to inflammatory cytokines, is likely to increase the conversion of testosterone to oestradiol.

### Strengths and limitations

The advantages of this study lie in the study design, which included cohort studies, cross-sectional studies, and case-control studies. To the best of our knowledge, our meta-analysis comprising 22 studies is the largest and first meta-analysis to evaluate the relationship between sex hormone levels and COVID-19. There are, however, also a number of disadvantages. First, the present meta-analysis had substantial heterogeneity across studies, which might be due to differences in study design and inconsistencies in baseline characteristics. Second, none of the studies were RCTs, so any causal pathway conclusions should be treated with appropriate caution. Third, many factors may have an impact on sex hormone levels, including genetic polymorphisms, age, other health conditions, sun exposure behaviour, and season. We thus cannot rule out that potential risk factors might have influenced our results. Finally, there is no specific cut-off point for sex hormone levels, which is important for clinical relevance. In the future, we will consider additional confounding factors to obtain more reliable and repeatable results.

## Conclusion

Overall, this study demonstrated that changes in sex hormone levels, such as decreased T/LH, FSH/LH, and SHBG levels and elevated LH, and E_2_/T levels, were strongly correlated with the severity and prognosis of COVID-19. We suggest that clinicians should be aware of the changes in sex hormone parameters of COVID-19 patients and seek guidance for treatment.

## Data availability statement

The original contributions presented in the study are included in the article/supplementary material. Further inquiries can be directed to the corresponding author.

## Author contributions

JinZ coordinated the study. ZC had the idea for the study, along with JiaZ, YJ and JinZ, contributed to the study design, literature search, figures, statistical analysis, data synthesis of outcomes and drafted and edited the final paper. All authors critically revised the report. All members have confirmed and agreed to submit the manuscript.

## Funding

This work was supported by grants from the National Natural Science Foundation of China (82070807, 91749118, 81770775, 81730022), the Planned Science and Technology Project of Hunan Province (2017RS3015) and National key research and development program (2019YFA0801903, 2018YFA2000100).

## Conflict of interest

The authors declare that the research was conducted in the absence of any commercial or financial relationships that could be construed as a potential conflict of interest.

## Publisher’s note

All claims expressed in this article are solely those of the authors and do not necessarily represent those of their affiliated organizations, or those of the publisher, the editors and the reviewers. Any product that may be evaluated in this article, or claim that may be made by its manufacturer, is not guaranteed or endorsed by the publisher.
